# Predicting target–ligand interactions with graph convolutional networks for interpretable pharmaceutical discovery

**DOI:** 10.1038/s41598-022-12180-x

**Published:** 2022-05-19

**Authors:** Paola Ruiz Puentes, Laura Rueda-Gensini, Natalia Valderrama, Isabela Hernández, Cristina González, Laura Daza, Carolina Muñoz-Camargo, Juan C. Cruz, Pablo Arbeláez

**Affiliations:** 1 Center for Research and Formation in Artificial Intelligence, Universidad de los Andes, Bogotá, 111711 Colombia; 2 Department of Biomedical Engineering, Universidad de los Andes, Bogotá, 111711 Colombia

**Keywords:** Machine learning, Virtual drug screening

## Abstract

Drug Discovery is an active research area that demands great investments and generates low returns due to its inherent complexity and great costs. To identify potential therapeutic candidates more effectively, we propose protein–ligand with adversarial augmentations network (PLA-Net), a deep learning-based approach to predict target–ligand interactions. PLA-Net consists of a two-module deep graph convolutional network that considers ligands’ and targets’ most relevant chemical information, successfully combining them to find their binding capability. Moreover, we generate adversarial data augmentations that preserve relevant biological backgrounds and improve the interpretability of our model, highlighting the relevant substructures of the ligands reported to interact with the protein targets. Our experiments demonstrate that the joint ligand–target information and the adversarial augmentations significantly increase the interaction prediction performance. PLA-Net achieves 86.52% in mean average precision for 102 target proteins with perfect performance for 30 of them, in a curated version of actives as decoys dataset. Lastly, we accurately predict pharmacologically-relevant molecules when screening the ligands of ChEMBL and drug repurposing Hub datasets with the perfect-scoring targets.

## Introduction

The development of novel drugs with therapeutic potential is a challenging yet essential endeavor to ensure human welfare and actively confront health threats. This process is characterized by conventional cycles lasting up to 12 years and demanding costs of about 2.7 billion US dollars^1^, with declining compensation due to limited efficacy and safety issues during clinical trial stages^2^. In this regard, the likelihood of approval for small molecule candidates for the period 2011–2020 was only 7.5%^3^, with expenditures ranging from 800 million to 1.4 billion US dollars in unsuccessful clinical trials^4^. These large yet unfruitful investments are currently forcing pharmaceutical and related sectors to search for more efficient strategies in terms of research objectives and profitability.

To address these shortcomings, reverse pharmacology, also known as target-based screening, proposes more robust data-driven approaches to improve the identification of active molecules towards therapeutic biological targets^5^. This methodology starts by detecting malfunctioning proteins as therapeutic targets of specific diseases through animal models or interdisciplinary analysis of patients phenotype and genotype. Once the targets are selected, high-throughput screenings are performed to detect the best pharmacological candidates against those targets^6^. This approach’s main promise is the possibility to increase predictability and accuracy of drug screening processes by systematizing analyses over large pharmaceutical datasets, which results in lessening the need for laborious in vitro and in vivo experimentation, and a significant increase in the overall efficiency of the entire discovery process. In this regard, virtual screening emerges as the current standard for the prediction of interactions between small molecules and proteins^7^. Conventional methods in virtual screening mainly employ molecular docking techniques, but are still limited in accuracy and effectiveness, and often require expensive experimental testing for validation prior to market launching^8,9^. In consequence, there is still a large scope for improving screening processes, especially regarding biological compatibility prediction.

Recent works use deep learning (DL) techniques in broad applications of the molecular biology domain, whose understanding is critical for the development of medicine and the comprehension of the biological interactions during physiological processes. For example, the prediction of functions for programmable RNA switches^10,11^, the three-dimensional structure prediction of proteins^12,13^, the prediction between protein-protein interfaces^14^, and the discovery of structurally distinct novel antibiotics^15^ are tasks in which DL has enabled the extraction of useful data and helped reduce laboratory experimentation. As in the research topics mentioned above, the interaction between small molecules and proteins is essential in the development of medicine, and therefore, human welfare. The information provided by the analyses of these interactions, such as the functional units, the binding pockets, and interaction sites between drugs and targets, is crucial for the targeted development and design of pharmaceuticals. For this reason, it is of general interest to know both if there is a target–ligand interaction and how this interaction occurs.

In this perspective, DL methods might be able to play a pivotal role due to their ability to find and exploit patterns in large datasets and distill salient features that characterize effective biological interactions between cellular targets and small molecules, henceforth regarded as target–ligand interactions (TLIs)^16^. Therefore, we address one of the most common formulations for TLI understanding: the binary classification of active and non-active interactions between a ligand and a protein. This task substantiates the larger endeavor of the niche market for drug discovery and repurposing, whose size is projected to grow from 24.96 to 34.62 billion US dollars between 2020 and 2027^17^.

Understanding biological interactions of pharmacological molecules involves reasoning over their intricate structures and corresponding traits at the atomic level. This working objective stresses the importance of appropriate molecular structure representation. In this respect, previous approaches have employed two-dimensional molecular images built from one-hot embeddings over atoms, a general method to vectorize categorical features, and have been analyzed under convolutional neural networks (CNN)^18^ and recurrent neural networks (RNN)^19^ to predict TLIs. However, these contributions disregard molecular structure, as well as the target protein’s information. Alternatively^20^ and^21^ incorporate the 3-dimensional (3D) structural information of the ligand positioned in the target receptor to predict activity through CNNs. Nonetheless, this type of input complex needs additional pre-processing and previous knowledge of the 3D structure of the protein, its binding pockets and the ligand position within the pocket, which are not easily available. Given the non-Euclidean nature of chemical data, molecular graph representations enable a more accurate and explicit modeling of atom and bond spatial configurations. Moreover, the conjunction of graph representation and deep learning techniques provides a promising approach to model molecular structures, both of small molecules and proteins, while extracting features with relevant biological backgrounds^22,23^.

Despite recent success in improving TLI predictions, their interpretability is a recurrent issue to validate their translation into compelling medicine scenarios. This feature is particularly important since high-performance metrics fail to provide sufficient information for evaluating if the model is learning relevant biological and chemical data for pharmacological design. To circumvent this situation, attention methods have emerged as powerful tools to identify key substructures in both targets and ligands for the model’s predictions^23^. Zheng et al., for instance, implemented a multi-head attention algorithm for both target and ligand embeddings to visualize more easily where the model focuses to predict TLIs^24^. Although their method is able to identify real overlapping regions of interaction in both ligands and targets, they disregard structural information by using linear representations of the molecules as input.

In this work, we propose the application of graph convolutional networks (GCNs) for predicting TLIs, where graph-based representations of both molecules and target proteins are obtained from easily accessible simplified molecular-input line-entry system (SMILES) strings and amino acid sequences in FASTA format. We address the most common limitations of GCNs, such as depth limits due to vanishing gradients, over-smoothing and loss of spatial information, by building on the work of^25^ and^26^ in the context of TLIs. We harness their proposed method for extrapolating common CNN strategies (e.g., residual and dense connections, dilated convolutions) to GCNs, which they demonstrated capable of enabling the training of deeper networks for a variety of computer vision tasks. This contrasts with previous graph-based methods proposed for molecular property prediction where limited depth and small receptive fields are among their main limitations. For instance, DeepChem equipped with graph convolutional model from^27^, and ChemProp^15^ have up to six message-passing layers, and PotentialNet^28,29^ has 3 stages of graph spatial convolution. Accordingly, we expect that employing deeper networks with larger receptive fields will favor the learning of global chemical information and will be reflected on overall performance. Moreover, we propose a method for increasing the interpretability of our TLI predictions by training the networks with adversarially-augmented molecules. These, in turn, are inspired by adversarial examples, specialized inputs with small intentional feature perturbations that cause machine learning models to make false predictions^30^. These have gained increasing popularity over the past few years in the field of computer vision due to their ability to direct learning towards semantically-aligned features^31,32^. In particular, we introduce a biologically-bounded gradient-based method to generate adversarial molecule augmentations, which adapts gradient-based edge deletion methods previously proposed for adversarial examples in graph data^33^ while preserving biological consistency and essential class features in molecular graphs.

**Figure 1:**
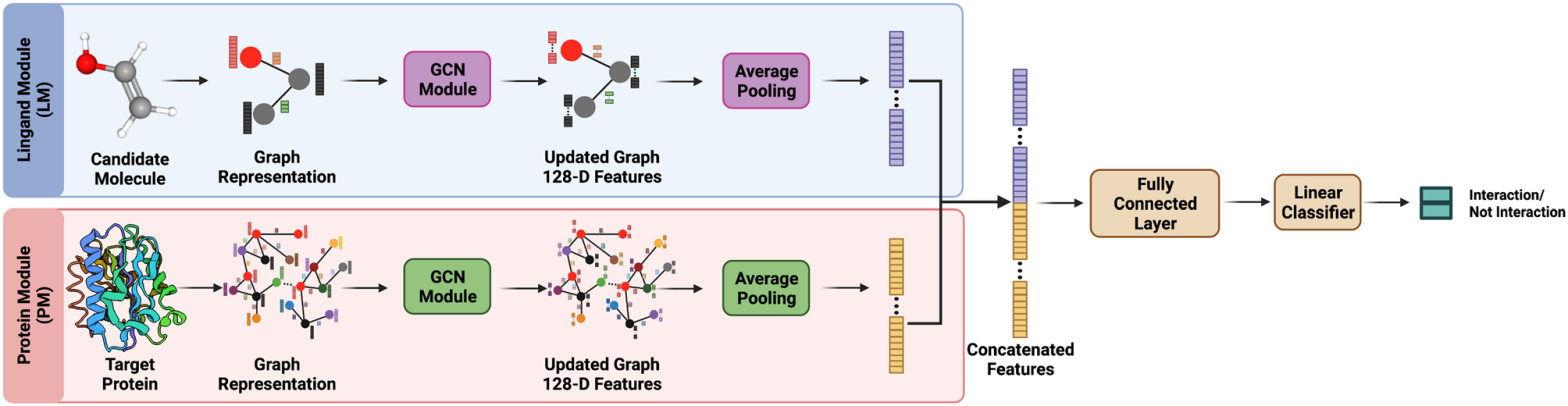
PLA-Net workflow. Schematic representation of a PLA-Net model for predicting interactions between small organic molecules and one of the 102 target proteins in the AD dataset. Graph representations of the molecule and a given target protein are generated from SMILES and FASTA sequences and are used as input to the Ligand Module (LM) and Protein Module (PM), respectively. Each module comprises a deep GCN followed by an average pooling layer, which extracts relevant features of their corresponding input graph. Both representations are finally concatenated and combined through a fully connected layer to predict the target–ligand interaction probability. Created with BioRender.com.

Our network, henceforth termed protein–ligand with adversarial augmentations network (PLA-Net), comprises two modules that separately extract information from proteins and ligands, thereby learning optimal representations for further compatibility analysis (Fig. 1). Our contributions are three-fold: (1) we model the spatial configuration of both the target protein and the ligand through bidirected graphs, (2) we integrate relevant chemical/primary structure information from proteins for TLI prediction, and (3) we propose a gradient-based method to compute adversarial molecule augmentations that preserve relevant biological backgrounds and improve both interpretability and overall performance. We train PLA-Net models for 102 pharmacologically-relevant protein targets and establish the new state-of-the-art, outperforming the previous one^19^ by a large margin of 19.8% on mean average precision (mAP) in a curated version of the actives as decoys (AD) dataset. Moreover, we perform a virtual screening between molecules of two large datasets (ChEMBL^34^, and Drug Repurposing Hub^35^) with the perfect-scoring targets, and corroborate that our method accurately predicts experimentally validated TLIs. We also demonstrate that PLA-Net is able to identify relevant substructures in molecules reported to interact with proteins of clinical interest. Both the outstanding performance and the interpretable results position PLA-Net as a promising starting point to integrate deep learning methods into drug discovery pipelines.

## Results and discussion

### PLA-Net considerably outperforms state-of-the-art models in the proposed benchmark

**Figure 2:**
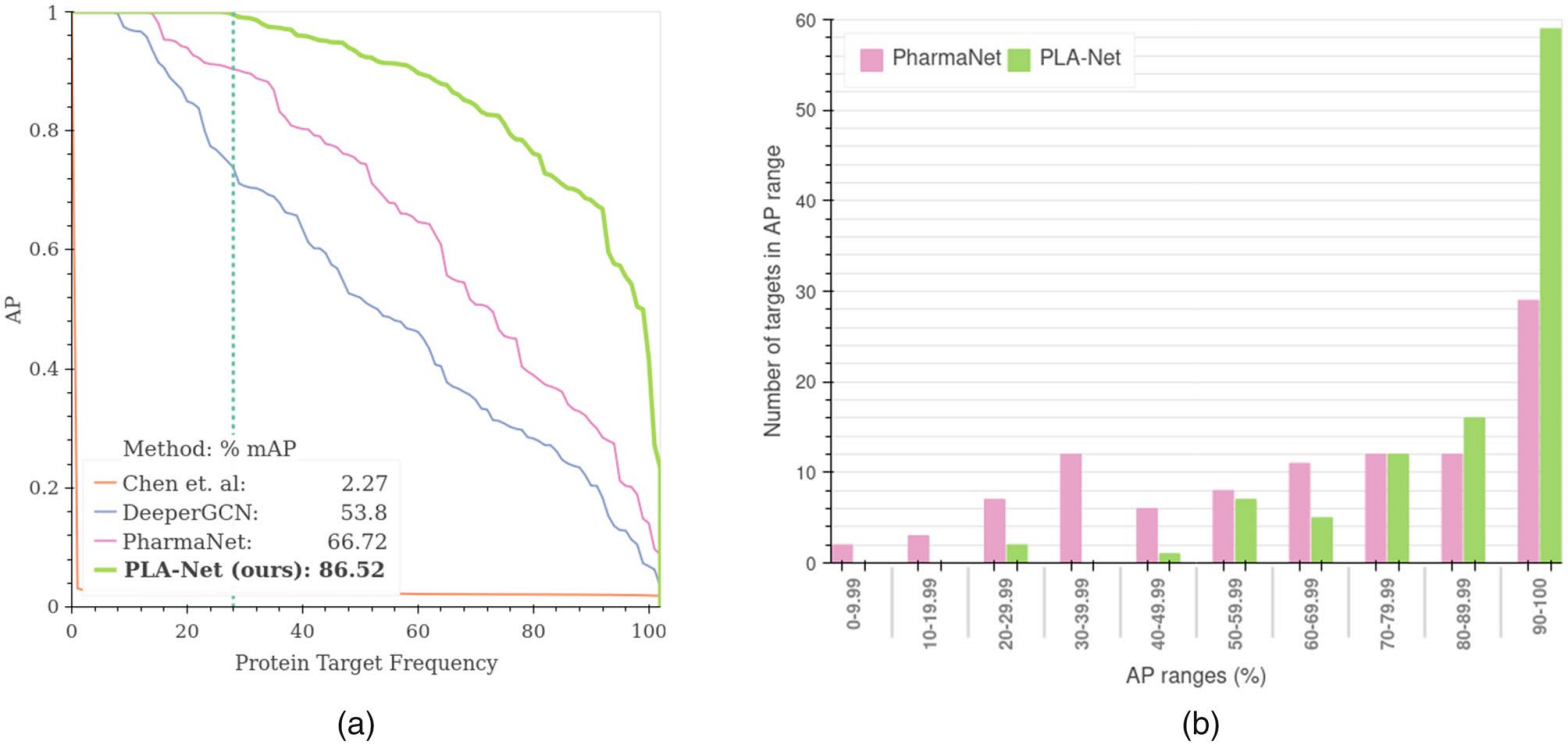
Comparison with state-of-the-art methods trained for TLI prediction in the proposed benchmark. (**a**) Performance distribution curves comparing our model (PLA-Net) with state-of-the-art methods. For each model, we show the number of binary models that achieve a TLI prediction performance greater than or equal to a specific AP value. (**b**) We compare the performance distribution of the 102 targets in PLA-Net with that of the current state-of-the-art (PharmaNet)^19^, showing that PLA-Net consistently improves the AP metric of the majority of the targets, with 59 targets with performance between 90 and 100% versus 29 in PharmaNet. Furthermore, PLA-Net achieves perfect performance for 30 targets with high clinical interest.

Figure 2a shows that PLA-Net significantly improves TLI prediction and outperforms by a large margin TLI state-of-the-art methods^19,36^ and DeeperGCN^26^ trained for this task. Besides increasing over 19 points in mAP from the highest-performing method^19^, the performance distribution of PLA-Net is superior than in the referred methods. Figure 2b shows that PLA-Net considerably shifts the performance histogram of the 102 models to the right, when compared to the current state-of-the-art method (PharmaNet)^19^. The models for 47 targets in PLA-Net achieve performances above 95% AP, which contrasts with only 18 targets in the case of PharmaNet. Moreover, 30 of these models achieve perfect scores, compared to only 13 in PharmaNet^19^. This suggests that the method and training curriculum proposed may enable a more explicit modeling of TLIs than previous works. The performance of all PLA-Net models is listed in Supplementary Table 1.

Other descriptors such as number of amino acids (AAs), intraclass similarity and number of actives/decoys per target are also included for comparison. However, we found no apparent correlation between these descriptors and the performance of PLA-Net models. The size of protein targets varied between 100 and 1434 AAs, with large and small proteins having both high and low performances. For instance, NOS1 (1434 AAs) and IGF1R (1367 AAs) achieve performances of 98.89% and 23.31%, while PA2GA (144 AAs) and TRY1 (247 AAs) achieve performances of 100% and 50.02%, respectively. Most importantly, within the 30 perfect-scoring targets there were both small proteins, such as FKB1A (108 AAs) and PA2GA (144 AAs), and large proteins, such as ROCK1 (1324 AAs) and ITAL (1170 AAs). Likewise, high and low performing protein models exhibit similar intraclass similarity distributions, which vary between 0.78 and 0.93. This suggests that performance differences may be merely a result of varying difficulties between TLI tasks.

### Graph representations capture more relevant molecular information than linear representations

To exploit the information-rich graph representations proposed, we optimized the parameters of our LM (e.g., optimizer, scheduler, average pooling method, depth) to boost its discriminative power. Ablations were performed over 15 representative targets, selected according to varying levels of difficulty are shown in Table 1. We compared the performance of the optimized LM with a classical machine learning technique (i.e., a random forest), and the current state-of-the-art approach (i.e., PharmaNet)^19^. The random forest takes as input a 6027 feature vector, extracted following^37^’s definition of atom pair descriptors, while PharmaNet takes as input the SMILES strings for each molecule. Since these methods were originally trained in a multiclass setup, we trained our LM in the same setting for fair comparisons. Additionally, we retrained PharmaNet in our proposed binary classification setup to compare their performance in both cases. We ablated the protein information from our model to ensure a fair comparison between methods.

**Table 1:** Ligand module optimization. To optimize LM module, we performed ablations over 15 representative targets, which were selected according to varying levels of difficulty.

Ligand module ablation	mAP (%)
**Number of Message Passing Layers (Depth)**	
7 Layers	76.09
15 Layers	75.92
**20 Layers**	**83.13**
50 Layers	81.41
**Aggregation Function**	
PowerMean Function	77.95
**Softmax Function**	**83.13**
**Graph Pooling Method**	
Max	77.95
Sum	74.98
**Mean**	**83.13**
**Features Hidden Size**	
64	62.08
**128**	**83.13**
256	82.46

Top performances in each setting are highlighted in bold.

Table 2 shows that our LM outperforms the random forest classifier and PharmaNet models in both multiclass and binary setups. In particular, the LM trained in the binary setup achieves performances over 5% and 10% higher than these competing models. These results highlight the importance of structural information granted by graph representations of ligands, especially when training binary models for predicting TLIs with each target. Even though training one model is inherently more efficient, this training time is counteracted by the benefit of models achieving highly accurate predictions for specific therapeutic targets with clinical relevance. Accordingly, all experiments were performed in the binary setup.

**Table 2:** Graph representation ablation study. LM: Ligand Module. AP: Atom Pair.

Method	Multiclass benchmark	Binary benchmark
Random Forest (AP descriptors)^38^	63.68	77.82
PharmaNet^19^	66.7	72.56
LM	72.29	82.58
PLA-Net	–	**86.52**

Best performance in bold.

### Including protein information improves TLI predictions.

**Figure 3:**
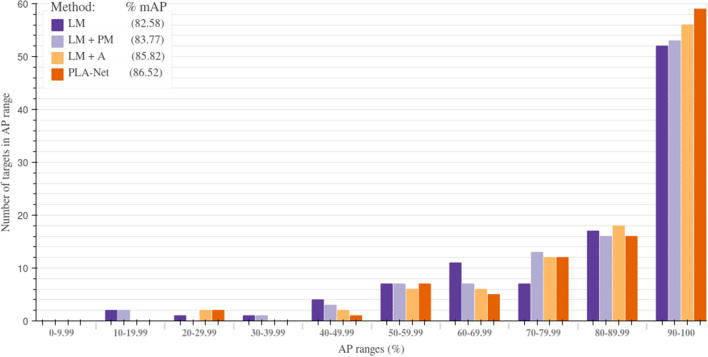
Performance distribution of PLA-Net training stages. The performance of individual targets shows a marked tendency towards high and perfect mAP scores (90–100%) as the training curriculum progresses. In particular, LM $+$ PM and augmented LM (LM $+$ A) show a clear improvement in performance distribution with respect to LM, and this is further improved when combining the information extracted by each in PLA-Net. Best viewed in color.

**Figure 4:**
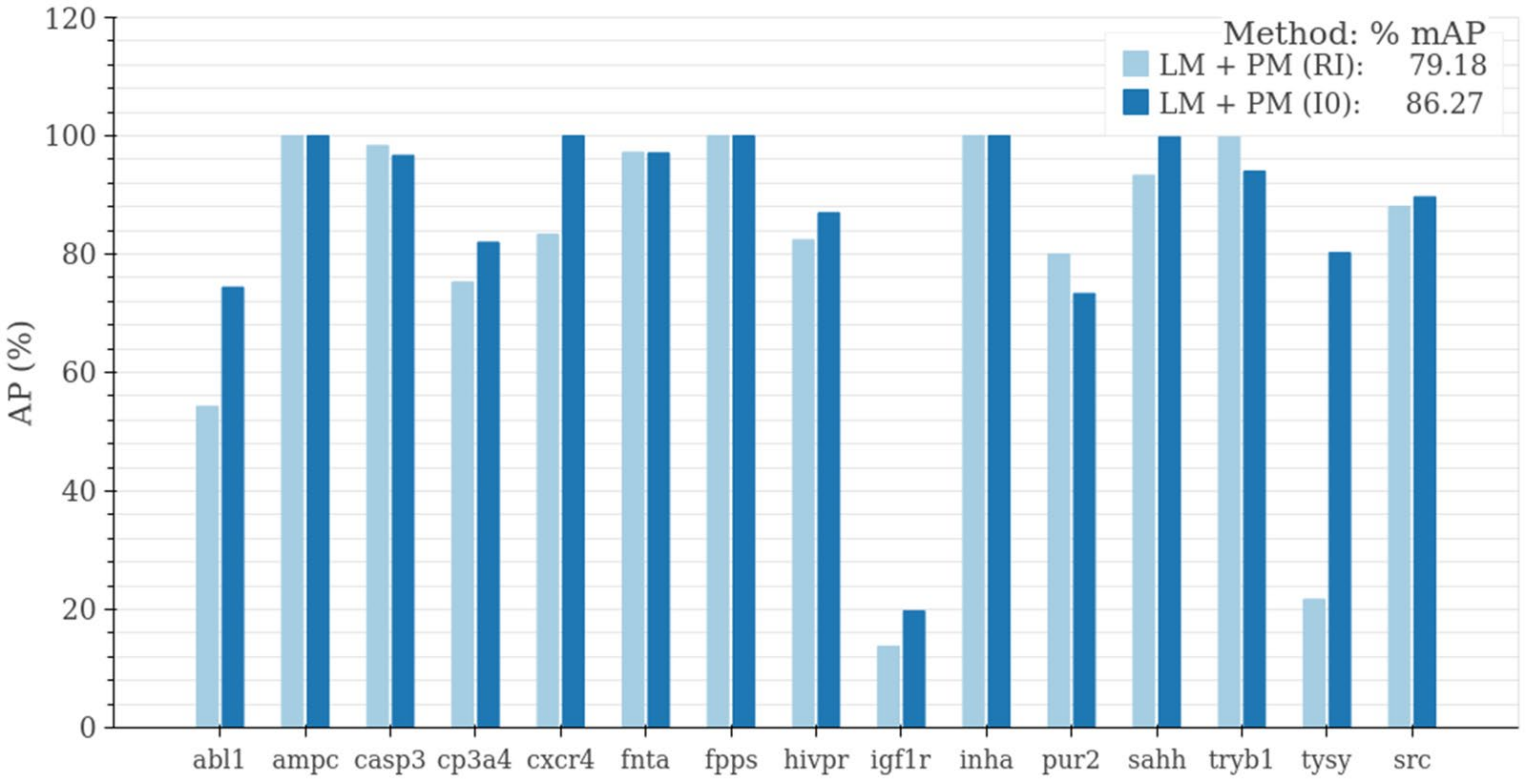
Initialization of protein contribution during LM $+$ PM training. Zeroing the linear classifier’s weights that correspond to the protein contribution at the onset of the training (I0) substantially improves the performance compared to a random initialization of the protein contribution (RI). We measured performance in mAP for 15 representative targets.

Since the information of target proteins is fundamental when assessing a TLI, we hypothesized that its inclusion would enhance the classifier’s discriminative power between actives and decoys. In particular, we expected that training the PM jointly with the pre-trained LM would promote the former to learn features specifically relevant to the target protein’s interaction with its ligands. As expected, including protein information increases the performance of 66.7% of the 102 models and improves the mAP metric by 1.19 points when compared to the LM alone (Fig.  3). The incorporation of target protein information during LM + PM training was optimized by including a learnable parameter that multiplied the fully connected layer weights associated with the protein information, thus controlling their contribution. We also show that initializing this multiplier as zero increases the performance of our models by allowing a smoother incorporation of this data when compared to random initializations (Fig. 4). This ablation was performed over the models for the same 15 representative targets selected above and resulted in an increase of 7.08 points in the mAP metric with respect to randomly initialized weights.

To verify that incorporating protein information is effectively improving the predictive power of our model, instead of just prolonging training, we also trained the 102 models replacing the output of the PM by a constant 128-D vector of ones. This ablation resulted in an average performance of 82.45% mAP, which is slightly lower than when training the LM alone. This was an expected result considering that the vector of ones is not contributing additional information about the TLI, which forces the model to focus mainly on the information provided by the LM. Moreover, it corroborates that the learned representations by the PM are contributing valuable information for the TLI prediction.

### Adversarial data augmentations improve model performance and interpretability

We propose the inclusion of adversarial molecule augmentations specifically tailored to harness the model’s weaknesses during training. Accordingly, we intend to help the model learn semantically-aligned features of the active molecules for each target and effectively discriminate them from decoys. Following the augmentation process described in “Adversarial data augmentations improve model performance and interpretability” section, we generated adversarial examples of the active molecules to each target at each training stage. This augmentation regime improved the average performance of the LMs alone by 3.25 points, increasing the performance of 55% of the 102 models and maintaining the performance of 40% (Fig. 3). In particular, 13 of the models increased by more than 10 points in AP. Supplementary Fig. 1 shows that the LM and the augmented LM of the 15 representative targets described above converge early in training, which verifies that the observed increase in performance was not merely due to the increase in computation. Overall, this suggests that the generated active molecules in each training stage were sufficiently similar to experimentally-proven ligands to maintain class consistency but also sufficiently different for the model to learn relevant features that it failed to learn with the original dataset.

**Figure 5:**
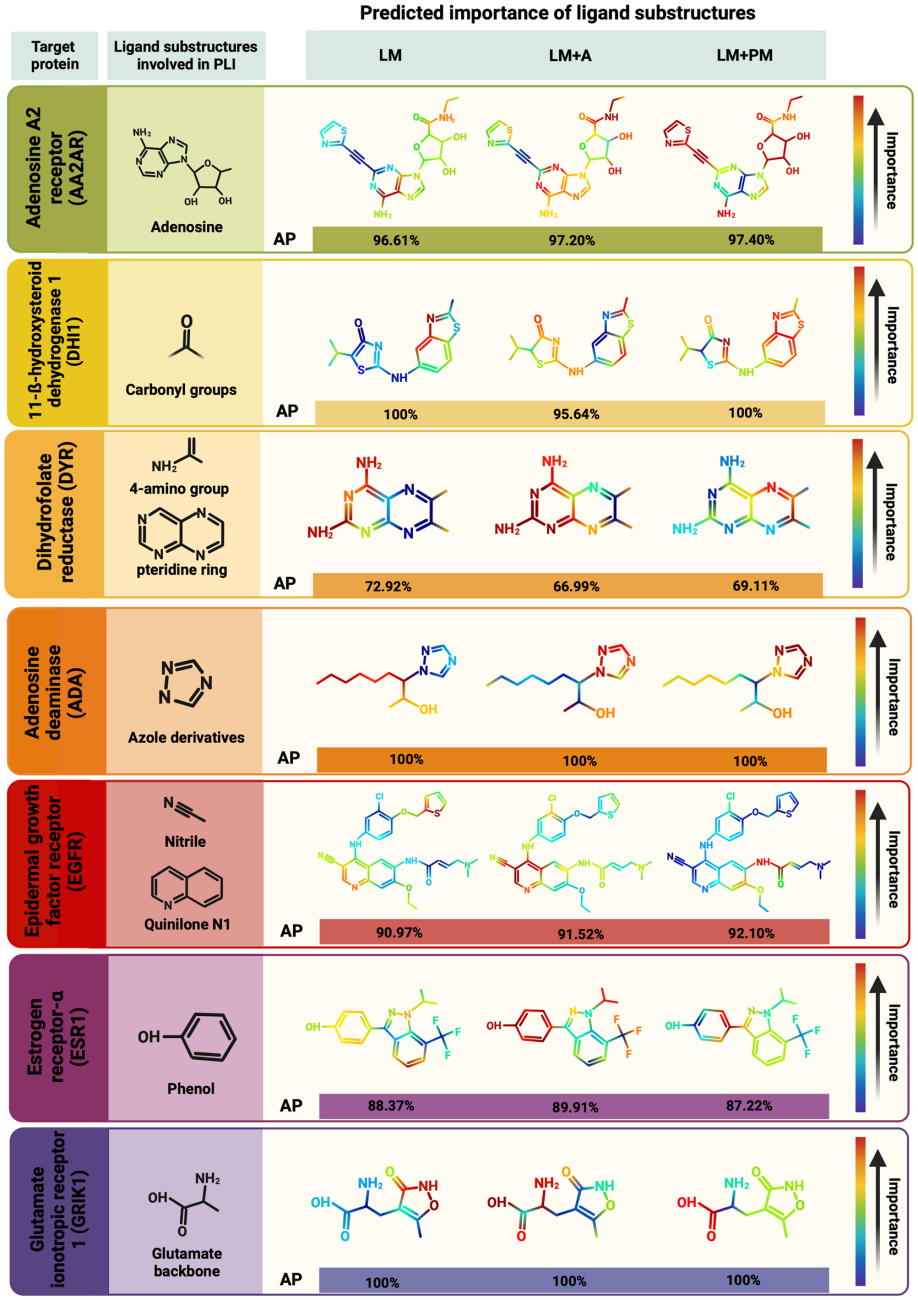
Salient feature maps of ligands during PLA-Net training stages. Salient feature maps predicted by the LM trained only on original molecules (LM), the LM trained with adversarial augmentations (LM $+$ A), and the LM and PM jointly trained (LM $+$ PM) for representative ligands of 7 protein targets. The average precision (AP) of each model is presented below their respective feature map and TLI-relevant substructures are shown to the left. All of these substructures have been previously identified through experimental and/or molecular docking analyses between the shown ligand and its respective target protein^39–44^. The predicted importance of ligand substructures significantly shifts at each training stage despite small changes in AP. The augmented LM achieves predictions that best align with substructures of natural ligands that have been previously reported to participate in TLIs. Created with BioRender.com.

To assess the effect of the proposed training schemes over the interpretability of our model, we conducted a gradient-based analysis to highlight the learned molecular salient features in each scenario. The atoms with the lowest gradient within the molecular graphs of active molecules were interpreted as the most important for the TLI prediction, due to their highest contribution towards minimizing the loss function. To exemplify this analysis, Fig. 5 compares the predicted importance of ligand substructures when training with augmented molecules (LM $+$ A), with protein information (LM $+$ PM) or with neither (LM). We also show substructures that have been previously reported to participate in the TLI of that specific ligand with its target to validate the models’ predictions. Notably, LM $+$ A and LM $+$ PM exhibit marked attention shifts towards localized substructures that have been previously reported to be involved in the respective TLIs. For instance, adenine and ribose substructures are predicted to be the most important for TLIs with the adenosine A2 receptor (AA2AR) by LM $+$ A and LM $+$ PM models, respectively, which coincides with the importance of adenosine substructures commonly found in AA2AR ligands^39^. In contrast, the pristine LM model fails to capture this information despite achieving an AP only 1% lower. Similarly, the glutamate backbone in ligands for the glutamate ionotropic receptor (GRIK1) is partially or completely highlighted when employing either of these training schemes, while it is given the least importance when training without them (LM). Considering that the pristine LM model for TLI prediction with GRIK1 already achieves a perfect-score, this suggests that the additional information included during LM $+$ A and LM $+$ PM training is shifting model training towards learning interpretable features beyond improving performance. This statement also holds true for other targets such as the adenosine deaminase (ADA), dihydrofolate reductase (DYR) and the epidermal growth factor receptor (EGFR), although much more prominently for adversarially-augmented models. In these cases, LM $+$ A is able to focus on relevant substructures whose interaction with the target’s binding pocket has been previously described with molecular docking analyses (e.g., azole groups for ADA^40^, 4-amino groups and pteridine rings for DYR^41^ and quinilone N1 and nitrile groups for EGFR^42^). Moreover, the importance of specific functional groups directly involved in the function of enzymatic targets is also best captured by LM $+$ A models, such as carbonyl groups in the ligands for 11-b-hydroxysteroid dehydrogenase 1 (DHI1), which are reduced into hydroxyl groups upon their interaction with DHI1^43^.

However, the attention shifts induced by the inclusion of target protein information in some LM $+$ PM models was not as fruitful as when training with augmented molecules. Phenol groups in ligands for the estrogen receptor alfa (ESR1)^44^, for example, lose importance in LM $+$ PM models despite being directly implicated in the TLI of this ligand with its receptor. Moreover, the importance of the N2 and nitrile groups of quinilone^42^ is completely lost when training with the protein information of EGFR, even though the AP achieved by the LM $+$ PM model for this target is higher than that of the LM model. Overall, these results suggest that the observed increase in interpretability is not merely a result of the increase in model performance, but that the type of additional information does play a role in what the models are learning. In particular, the additional information provided by the generated adversarial molecules appears to be the most useful in directing training towards the learning of semantically-aligned features, which is one of the major challenges in the training of generalizable neural networks^45^.

### PLA-Net’s perfect scoring models predict pharmacologically-relevant TLIs on drug repurposing and ChEMBL databases

**Figure 6:**
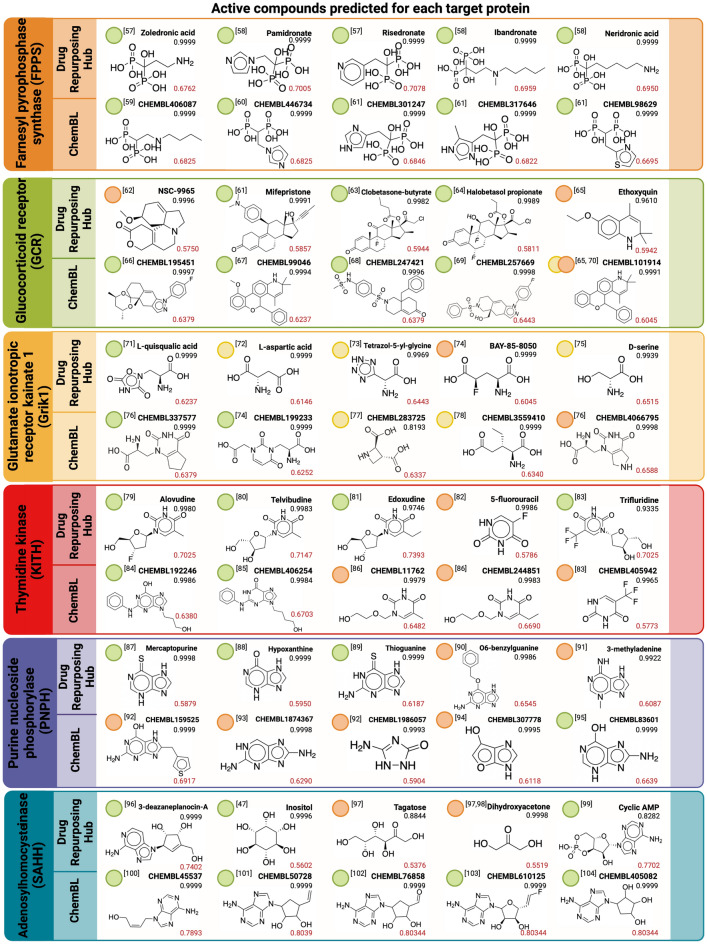

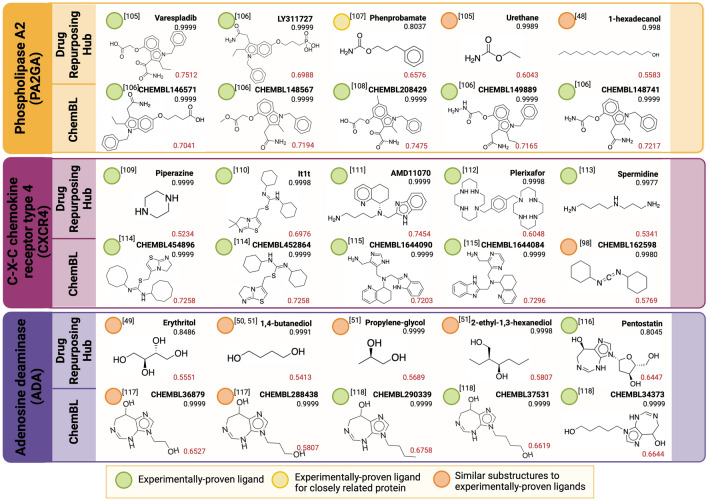
PLA-Net’s pharmacologically-relevant TLI predictions on the Drug Repurposing and CHEMBL databases. From each database, were selected five molecules predicted as active with high probability for nine pharmacologically-relevant targets. The name and prediction probability for each molecule are shown in their upper right corner. The mean Rogot–Goldberg similarity between each molecule and the active molecules of the corresponding training set is shown in red in their lower right corner. The mean Rogot–Goldberg similarity between each molecule and the active molecules of the corresponding training set is shown in red in their lower right corner. Molecules’ activity towards each target was corroborated with previous literature reports^57–118^ . Green label: experimentally-proven active molecule for the respective target. Yellow label: experimentally-proven active molecule for protein closely related to the target of interest. Orange label: not experimentally-proven, but with relevant substructures present in experimentally-proven active molecules for the target. Created with BioRender.com.

To validate that PLA-Net models were able to make reasonable predictions in previously unseen data, we perfomed a high-throughput virtual screening for pharmacologically-relevant molecules on Drug Repurposing and ChEMBL databases. In particular, we predicted TLIs with 9 protein targets that achieved perfect scores during training and are closely related to different pharmacological applications (Table 3). We validated the top predictions of our models by manually corroborating them with previous literature reports. Figure 6 shows five molecules from each database that PLA-Net predicted to interact with each target with a high probability. Red scores correspond to the Rogot–Goldberg similarity between the predicted ligands and the active molecules in the training set. Green labeled molecules have been previously reported as active for the target of interest, yellow labeled molecules have been reported as active for a closely related protein, and orange labeled molecules have not been reported but exhibit relevant substructures present in active molecules for the same target.

**Table 3:** Perfect-scoring targets with pharmacological applications used for virtual screening in the drug repurposing Hub and ChemBL databases.

Acronym	Target protein	Pharmacological application
FPPS	Farnesyl pyrophosphase synthase	Anticancer and Antimicrobial
GCR	Glucocorticoid receptor	Anti-inflammatory
GRIK1	Glutamate ionotropic receptor kainate 1	Neurophysiological
KITH	Thymidine kinase	Anticancer and Antimicrobial
PNPH	Purine nucleoside phosphorylase	Anticancer
SAHH	Adenosylhomocysteinase	Anti-inflammatory
PA2GA	Phospholipase A2	Anticancer
CXCR4	C-X-C chemokine receptor type 4	Antibacterial and Anticancer
ADA	Adenosine deaminase	Immunomodulation

PLA-Net was able to accurately predict, for all target proteins, multiple molecules that have been previously reported as active towards each target, as well as molecules that follow consistent structural patterns. All molecules predicted as active for farnesyl pyrophosphate synthase (FPPS), for example, include biphosphate groups within their structure, which is a recurrent hallmark of FPPS ligands given the active participation of this group in their interaction with FPPS^46^. Accordingly, all shown predictions have been previously reported as FPPS inhibitors. Moreover, the relatively low fingerprint similarities between the predicted ligands and the training set ligands (0.67-0.71) suggest that, despite the consistent appearance of biphosphate groups, side-chain elements can vary widely. This demonstrates that the model is capable of prioritizing the presence of biphosphate groups and ignoring side-chain differences. Similarly, most molecules predicted as ligands towards PNPH, the purine nucleoside phosphatase, contain a purine substructure that correlates well with the enzyme’s function. In turn, all molecules whose interaction has not been reported are either substructures of reported molecules or have slight ramification changes of known active molecules. This pattern was consistently observed with other targets such as the glucocorticoid receptor (GCR), the thymidine kinase (KITH) and the adenosylhomocysteinase (SAHH). In these cases, steroid backbones, thymine substructures and adenine substructures were present in most predicted ligands, respectively. Notably, despite these consistent structural patterns, many experimentally-validated predictions for these targets also exhibit low similarities with active training molecules (0.56–0.59), which again suggests that these models are capable of recognizing consistent substructures within heterogenous backbones.

In contrast, some predicted molecules for targets such as SAHH, adenosine deaminase (ADA) and phospholipase A2 (PA2GA) showed no clear structural pattern, yet they still fit into one of two categories: (1) have been reported as active towards the protein or closely related proteins, or (ii) are substructures of active molecules for the target protein. In the particular case of SAHH, the predicted molecule Inositol has been experimentally proven as active towards this protein^47^, but its structure largely differs from the other experimentally validated predictions and active ligands from the training set (0.5602 average fingerprint similarity). Similarly, the predicted active molecule for PA2GA, 1-hexadecanol, is a substructure of its experimentally-proven ligand 2-Ethylamino-1-hexadecanol^48^, which fails to exhibit the purine-based structures observed previously in other experimentally-proven ligands for this target. The same trend is observed with the alcohol derivatives predicted for ADA, which are substructures of other previously reported ligands^49,50^ and their presence has shown to favor TLI occurrence^51^. This suggests that our model is able to learn relevant substructures from heterogeneous molecule sets and is not merely memorizing single structural patterns. Further evidence of this finding can be found by observing that several experimentally proven ligands for the C-X-C chemokine receptor type 4 (CXCR4) and the glutamate ionotropic receptor kianate 1 (Grik1) were accurately predicted, despite their marked structural differences and no apparent common backbone (0.52–0.63 fingerprint similarity with training sets). The observed discriminative power correlates well with the marked heterogeneity in the training sets used for these two targets, which comprise a wide array of molecules with divergent structural backbones and limited congruencies. For instance, although all Grik1 ligands coincide in a terminal glutamate-derived functional group, the large backbones differences between them are well captured within the dataset (Supplementary Fig. 2). Accordingly, although some predicted ligands are closely respresented by active molecules in the training set (e.g., L-quiscalic acid, CHEMBL4066795), the others that are not can still be accurately identified (e.g., CHEMBL199233, tetrazol-5-yl-glycine, CHEMBL283725). Furthermore, PLA-Net’s prediction scores are significantly higher than the similarity score, highlighting the advantage of our method over canonical fingerprint analysis. Overall, these highly accurate predictions validate that PLA-Net is effectively learning underlying structural hallmarks that dictate TLIs with protein targets. This is of special clinical relevance given their close involvement in numerous diseases such as cancer, chronic inflammation, neurodegeneration, and microbial infections.

## Conclusion

In this work, we propose PLA-Net, a two-module deep GCN to tackle TLI prediction in a curated version of the AD dataset. Our method merges rich information extracted from ligand and protein graphs utilizing deep GCN modules. Additionally, we propose a method for generating adversarial molecule augmentations that preserve biologically relevant backgrounds and show that their inclusion during training improves our model’s performance and interpretability. Accordingly, PLA-Net not only becomes the new state-of-the-art in TLI prediction, but allows a more comprehensive analysis of the underlying features dictating TLIs. Moreover, its highly accurate TLI predictions with molecules unseen previously by the network and extracted from large and unannotated databases brings us one step closer towards interpretable pharmaceutical discovery.

## Methods

### TLI benchmark

The Database of Useful Decoys Enhanced (DUD-E) is widely employed to benchmark approaches that predict TLIs^52–54^. DUD-E contains 22,886 experimentally verified active compounds towards 102 proteins of clinical interest listed in Supplementary Table 1. Some of these proteins are related to chronic diseases of significant clinical interest such as hypertension (RENI), HIV (CXCR4), cancer (FPPS), and Parkinson’s disease (COMT). Additionally, for each active molecule, DUD-E contains 50 non-active compounds (decoys) with similar physicochemical properties but different topologies. This unbalanced data distribution replicates the real scenario of finding an active compound against a huge variety of decoys.

However, Chen et al.^36^ proved that DUD-E’s selection criteria for the decoys follows a pattern that makes them easily distinguishable from the active molecules. Conversely, they proposed the Actives as Decoys (AD) dataset, in which the decoys are selected from the active molecules of other protein targets. In particular, the decoys for a protein are selected by performing molecular docking between that protein and the active compounds from the other 101 proteins. The TLI is then ranked based on the predicted binding energy. The top-50 molecules of each of the 101 targets are selected to be the decoys of that target protein. This process is repeated for all targets to create the decoy dataset.

Nonetheless, we noticed that some molecules in the DUD-E dataset are labeled as active for more than one protein target and, therefore, the decoy selection process of the AD dataset causes some molecules to be simultaneously labeled as active and decoy for the same protein. In addition, most decoys for the same protein are repeated. Since this contradictory and redundant information might detrimentally lead to a bias during model training, we removed the repeated samples (546,412 molecules) and variably labeled samples (37 molecules) for each target, which represent 52.29% of the original data. We also ensure that active compounds of the targets are the same as those experimentally validated in DUD-E. Subsequently, we separated the curated version of the AD dataset into training and testing sets, comprised of 90% and 10% of the compounds for each protein, respectively. Next, we performed a four-fold cross-validation with the training subset. Protein information for the 102 protein targets in the AD dataset was obtained by compiling their FASTA sequences from the Universal Protein (UniProt) repository.

We evaluated with the Average Precision (AP) metric and report the mean AP (mAP) of the predicted interactions with the 102 target proteins.

### Molecule representation

The molecular representation used by state-of-the-art methods in the AD Dataset, SMILES, fails to recapitulate the complexity of molecules mainly due to the absence of structural information that dictates interactions between atoms. Other methods rely on very complex 3D representations, which cannot be obtained for all protein-ligand pairs because they depend on experimental techniques such as X-ray crystallography and computational techniques such as molecular docking. In contrast, graph representations are well suited for capturing molecular information since they enable the reconstruction of atomic networks that preserve chemical and structural information. For this reason, we built the graph representations of both ligands and target proteins based on their atomic and bond configuration. The RDKit package was used to convert their respective SMILES and amino acid sequences in FASTA format into molecular graph representations (Fig. 1). Given a ligand or protein, its graph is represented as $\mathscr {G} = (\mathscr {V},\mathscr {E},X_v,X_e)$, where $\mathscr {V}$ denotes the set of atoms (nodes), $\mathscr {E}$ the set of bonds (edges), $X_v$ the set of atom features and $X_e$ the set of bond features. An atom feature vector $x \in X_v$ comprises nine properties of the atom $v \in \mathscr {V}$: atomic number, chirality, degree, formal charge, number of hydrogens, number of radical electrons, hybridization, aromaticity and ring membership. Similarly, we built a bond feature vector $x_{vu} \in X_e$ from three characteristics of the bond $e_{vu} \in \mathscr {E}$ between atom *v* and atom *u*: type of bond, stereochemistry, and conjugation. Each feature is represented as a one-hot vector and all one-hot feature representations are then concatenated to form the feature vector. The length of each one-hot vector is determined by the possible options for describing each feature, which are summarized in Table 4. Moreover, all bonds are assumed bidirectional, $X_{e_{vu}} = X_{e_{uv}}$. Accordingly, this graph representation enables the learning of both chemical and spatial distribution properties due to the node/edge feature vectors and edge configurations between nodes, respectively. Furthermore, this representation favors the learning of implicit 3D information that is inherent to the modeled molecules such as the stereochemistry of bonds, chirality, and aromaticity. Moreover, note that geometrically optimized 3D models of either ligands or proteins are not necessary for graph construction due to the ease of extracting all necessary information directly from SMILES and FASTA strings, making this process computationally inexpensive.

**Table 4:** Atom and bond features. Feature vectors are obtained using RdKit and OGB libraries, which describe the state of an atom and a bond within a molecule.

	Atom features
Atomic Number	1, 2, ..., 119
Chirality	Unspecified, Tetrahedral clockwise, Tetrahedral anti-clockwise, Other
Degree	0, 1, ..., 10
Formal Charge	− 5, − 4, ..., 4, 5
Number of Hydrogens	0, 1, ..., 8
Number of radical e$^{-}$	0, 1, ... , 4
Hybridization	Sp, Sp$^{2}$, Sp$^{3}$, Sp$^{3}$d, Sp$^{3}$d$^{2}$
Aromaticity	0, 1
Ring membership	0, 1

	Bond features
Type	Single, Double, Triple, Aromatic
Stereochemistry	None, Z, E, CIS, TRANS, Any
Conjugation	0, 1

### Architecture details

As shown in Fig. 1, ligand and protein graphs are analyzed separately by a GCN module, which extracts chemical and spatial information to obtain optimal representations for further TLI analysis. Each module comprises a deep GCN and an average pooling function, which outputs a 128-D vector representation for the molecular graph. Both optimized representations are concatenated, a linear layer merges the information, and a fully connected layer then classifies the ligand as an active or decoy.

Our GCN modules were adapted from^26^, which is a message-passing framework originally designed for molecular property prediction. Being $\mathscr {N}_v$ the set of neighbors of atom *v*, the message passing algorithm of the *l*th layer is described by message construction (Eq. 1), message aggregation (Eq. 2) and node update (Eq. 3) functions.

1 $$\begin{aligned}&\mathscr {M}_{vu}^{(l)} = \rho ^{(l)}(X_v^{(l)},X_u^{(l)},Xe_{vu}^{(l)}), u \in \mathscr {N}_v \end{aligned}$$

2 $$\begin{aligned}&\mathscr {M}_{v}^{(l)} = \zeta ^{(l)}(\{\mathscr {M}_{vu}^{(l)} | u \in \mathscr {N}_v\}) \end{aligned}$$

3 $$\begin{aligned}&{X_v}^{(l+1)} = \phi ^{(l)}({X_v}^{(l)},\mathscr {M}_{v}^{(l)}) \end{aligned}$$

where $\rho ^{(l)}$, $\zeta ^{(l)}$, $\phi ^{(l)}$ are all learnable and differentiable functions. The message construction function $\rho ^{(l)}$ is applied to the features of atom *v*, the features of its neighbor *u*, and their corresponding edge features $x_{vu}$ to obtain an individual message $\mathscr {M}_{vu}^{(l)}$ for each neighbor $u \in \mathscr {N}_v$. The message aggregation function $\zeta ^{(l)}$ takes as input the set of individual messages and outputs an aggregated message. In this case, $\zeta ^{(l)}$ is a learnable softmax function. Finally, $\phi ^{(l)}$ updates the node features of the *l-th* layer by adding the aggregated message $\mathscr {M}_{v}^{(l)}$ and passing it through a multi-layer perceptron.

As mentioned in^25^ and^26^, to enable the adequate training of deep GCNs this architecture employs two techniques inspired in CNNs: (1) pre-activation variants of residual connections, and (2) dilated aggregations that enlarge the receptive field. In the former, training proceeds by a change in the usual ordering of GCN components. In other words, instead of performing the graph convolution, followed by normalization, a ReLU layer and the addition of the residual connection, this architecture performs first the normalization and ReLU activation, followed by the graph convolution and the addition of the residual connection. In the latter, inspired by dilated convolutions in CNNs, a dilated graph is constructed after each message passing layer^55^. In particular, the network relies on a *Dilated k-NN* to find dilated neighbors at a *d* dilation rate. This operation returns the *k* nearest neighbors within the *kxd* neighborhood region by skipping every *d* neighbors.

Our modules consist of 20 message-passing layers, and final embedding size for nodes’ and edges’ features of 128.

**Figure 7:**
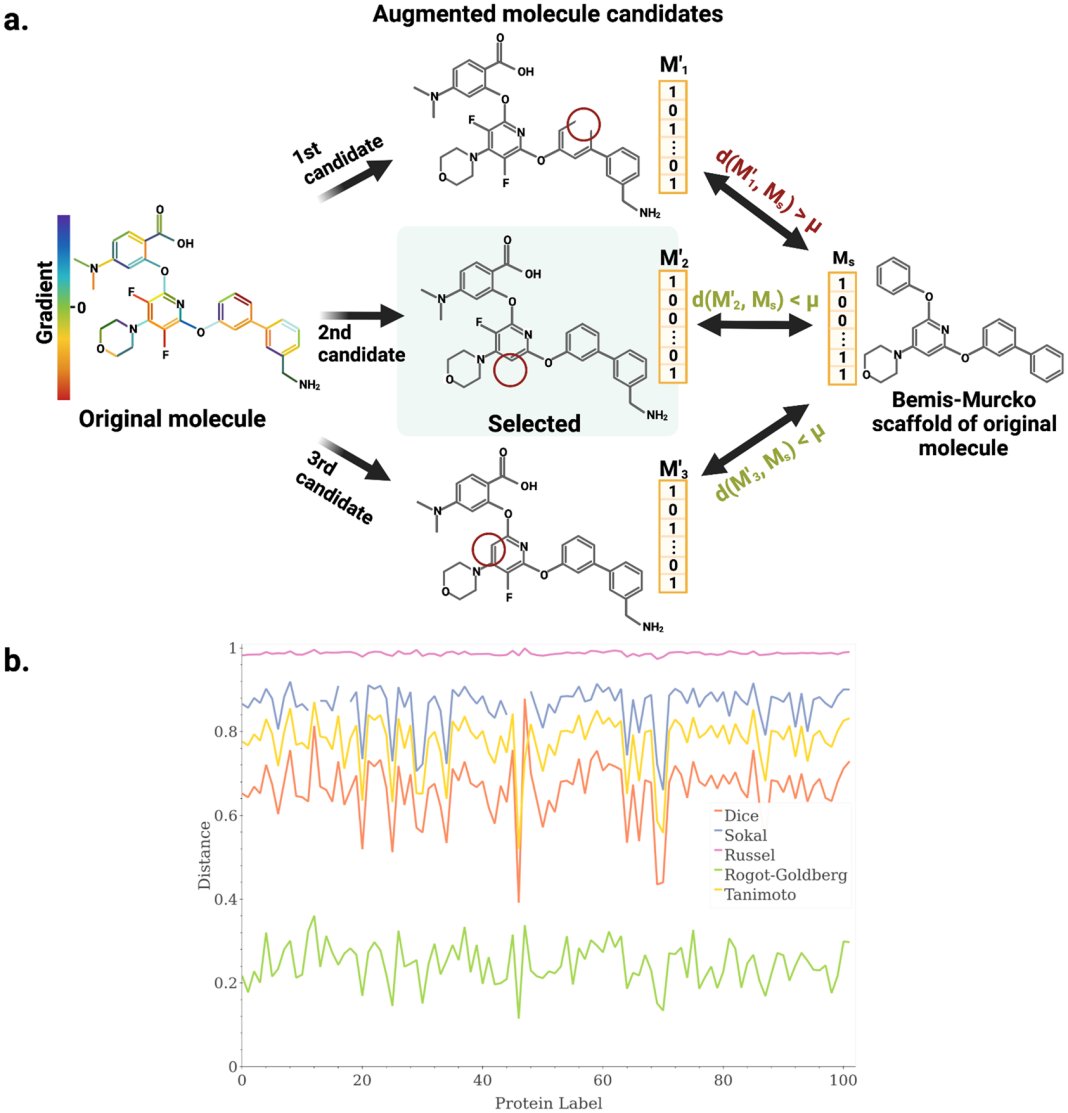
Adversarial augmentations. (**a**) Augmented molecules are generated through an edge-deletion process that selects the edge of the molecular graph to delete by following two criteria: (i) the deletion of the selected edge must generate an adversarial molecule whose distance to the Bemis–Murcko scaffold of the original molecule is less than a defined threshold ($\mu$) and (ii) the selected edge must have a negative gradient and the gradient magnitude must be maximal. (**b**) Comparison of intra-class distance as a function of different similarity metrics. We computed the distances between the Morgan fingerprints of molecules from a specific target class and of their corresponding Bemis–Murcko scaffolds to assess the average intra-class distance as a function of different similarity metrics. We selected the Rogot–Goldberg similarity descriptor due to its high performance for intra-class similarities. Created with BioRender.com.

### Adversarial data augmentations

To generate biologically relevant adversarial molecules, we propose a gradient-based method that modifies the edges of molecular graphs according to their contribution to the model’s outcome. In particular, we associate a binary coefficient with a value of 1 to each edge in the graph and multiply it with the feature vector of its corresponding edge during graph construction. Even though this multiplication maintains the original values of the feature vector and the computation of the molecule’s embeddings, the gradient of these binary variables after backpropagating through the model becomes crucial for determining which edges are contributing the most to the model’s predictions. In this context, the work by Dai et al.^33^ inspired us to delete the edge with the most negative gradient coefficient, that is, the one with the highest contribution in minimizing the loss function.

To ensure that the augmented molecule resulting from the deletion of the chosen edge preserves relevant class characteristics, we defined a molecular distance metric that bounds the edge selection process. We impose a distance constraint between the augmented molecule and the original molecule as a function of the similarity between the former and the Bemis–Murcko scaffold^56^ of the latter. This scaffold preserves crucial structural characteristics of a molecule by retaining backbone structures and eliminating side-chain elements^56^. We chose this scaffold as reference, instead of the original molecule, since we wanted to preserve molecular backbones in the augmented molecules. This is particularly important since modifying these structures may induce large conformational changes that could detrimentally impact the TLI under real physiological scenarios and, in turn, yield molecules that fail to preserve relevant class characteristics. Moreover, considering that Bemis-Murcko scaffolds were previously used to eliminate similar molecules while assembling the DUD-E dataset^52^, these scaffolds are highly suitable for molecule comparison.

The distance between augmented molecules and their respective scaffolds is computed over their Morgan fingerprints according to Eq. (4).

4 $$\begin{aligned} d(M_s, M') = 1 - RGS(M_s, M') \end{aligned}$$

where $M_s$ is the Morgan fingerprint of the Bemis–Murcko scaffold of the original molecule, $M'$ is the Morgan fingerprint of the augmented molecule, and RGS is the Rogot–Goldberg similarity between them. RGS was chosen above other fingerprint similarity metrics (e.g., Tanimoto, Dice, Sokal, Russel) since it minimizes the average distance between the fingerprints of molecules and their corresponding Bemis–Murcko scaffolds (Fig. 7b). To bound the described distance, we define the largest distance from the molecules to their corresponding scaffolds as a threshold $\mu$. If deletion of an edge causes the distance between the modified molecule and the scaffold of the original molecule to exceed the defined threshold, that edge is skipped to evaluate the next candidate, according to the negative gradient magnitude. If no edge with a negative gradient satisfies this condition, the molecule is skipped and no augmented molecule is retained. An example of this adversarial augmentation process is shown in Fig.  7a.

We generated augmented molecules for each batch and included them during training in addition to the original molecules. In this way, each batch included augmented molecules that exploit the strengths and weaknesses of the model at each training stage, tailoring the augmentation process to the model’s needs.

### Implementation details

PLA-Net models for each of the 102 target proteins were trained following a multi-step curriculum directed towards optimizing the molecule and protein representations such that their relevant information could be easily extracted and assembled. First, the Ligand Module (LM) was individually trained with only original molecules for 300 epochs and a learning rate (LR) of 5*e*−3. In this case, the 128-D feature embeddings outputed by the LM is directly passed through the classification layer since no protein information is included. A randomly-initialized Protein Module (PM) was then included and jointly trained with the previously trained LM for 20 more epochs to integrate protein information relevant to the TLI. This was done following the workflow described in Fig. 1. Simultaneously, another LM was trained from scratch, with both original and augmented molecules, for 300 epochs and a LR of 5e−4. Again, the outputed 128-D feature embeddings were passed directly through the classification layer as no protein information was included. Finally, the 128-D feature embeddings generated by the PM jointly trained in the first stage, and by the augmented LM of the second stage were concatenated and their extracted information was combined with the training of a fully connected layer and classification layer for 20 epochs and a LR of 5e−5. In this final step, the concatenated information is first transformed into a 128-D vector with the fully connected layer and then passed through a classification layer that yields the binary prediction for the TLI.

### Screening for drug discovery and drug repurposing

To validate the quality of our models’ predictions and propose new pharmaceutical candidates for our perfect scoring targets, we screened for TLIs on the ChEMBL (https://www.ebi.ac.uk/chembl/)^34^ and the Drug Repurposing Hub (https://clue.io/repurposing)^35^ databases. ChEMBL is a manually curated database with information for 15’504,604 bioactive molecules with drug-like properties^34^. Similarly, the Drug Repurposing Hub is a curated and annotated collection with 13,553 FDA-approved drugs, clinical trial drugs, and pre-clinically tested compounds developed with the aim of revealing new therapeutic targets for known drugs^35^.

We selected the models that achieved perfect TLI scores on the test set (i.e., models for 30 protein targets) for predicting TLIs with molecules in the Drug Repurposing Hub. Out of the selected models, we chose 11 to evaluate TLIs with ChEMBL molecules according to their clinical and therapeutic relevance. We filtered out from both databases the molecules whose SMILES could not be converted into a graph due to RDKit SMILES’-reading format. After this curation, we tested the 30 perfect-scoring models with 6,798 unique molecules of the Drug Repurposing Hub and the referred 11 models with 2’031,651 unique molecules of ChEMBL. Lastly, we selected the molecules with the 5 highest TLI scores for each tested target, after ensuring that those molecules were not considered for training the model.

## Supplementary Information

Supplementary Information.

## Data Availability

Code, implementation instructions, the curated AD dataset, PLA-Net weights and inference in drug repurposing and CHEMBL databases available at PLA-Net repository in https://github.com/BCV-Uniandes/PLA-Net.
