# Supplementary material for: Predicting target–ligand interactions with graph convolutional networks for interpretable pharmaceutical discovery
Source: Sci Rep. 2022 May 19;12:8434. doi: 10.1038/s41598-022-12180-x (PMC9119967; doi:10.1038/s41598-022-12180-x)
Supplement: Supplementary file 1 — Supplementary Information. [file 41598_2022_12180_MOESM1_ESM.pdf]

Supplementary Material: Predicting Protein-Ligand Interactions with Graph Convolutional Networks for Interpretable Pharmaceutical Discovery

Paola Ruiz Puentes[1,2,+], Laura Rueda-Gensini[1,2,+], Natalia Valderrama[1,2,+], Isabela Hernández[1,2], Cristina González[1,2], Laura Daza[1,2], Carolina Muñoz-Camargo[2], Juan C. Cruz[2], and Pablo Arbeláez[1,2,*]

[1]Center for Research and Formation in Artificial Intelligence, Universidad de los Andes, Bogotá 111711, Colombia
[2]Department of Biomedical Engineering, Universidad de los Andes, Bogotá 111711, Colombia
[*]corresponding.author@email.example
[+]these authors contributed equally to this work

Table 1. Model descriptors for 102 target protein.
Figure 1. Convergence plots per fold of the LM and augmented LM of 15 representative targets.
Figure 2. Comparison of PLI predictions on the Drug Repurposing Hub and CHEMBL data.

Table 1. Model descriptors for 102 target proteins. The acronym, name, number of amino acids (AAs), PLA-Net model performance in average precision, average Rogot-Goldberg similarity between active ligands, number of active ligands and number of decoys are shown for each of the 102 target proteins.

Acronym	Target Protein	# of AAs	AP	Similarity train-test sets	Active ligands	Decoys
ADA	Adenosine deaminase	363	1	0,848±0,027	92	4626
ADA17	ADAM17	824	1	0,902±0,028	531	4653
AMPC	Beta-lactamase	377	1	0,867±0,024	47	4662
COMT	Catechol O-methyltransferase	271	1	0,811±0,026	40	4693
CXCR4	C-X-C chemokine receptor type 4	352	1	0,954±0,026	39	4641
DEF	Peptide deformylase	169	1	0,853±0,027	101	4688
DPP4	Dipeptidyl peptidase IV	766	1	0,891±0,025	532	4680
FKB1A	FK506-binding protein 1A	108	1	0,934±0,024	110	4651
FPPS	Farnesyl diphosphate synthase	419	1	0,785±0,024	84	4676
GCR	Glucocorticoid receptor	777	1	0,888±0,025	257	4669
GLCM	Beta-glucocerebrosidase	536	1	0,848±0,025	53	4673
GRIK1	Glutamate receptor ionotropic kainate 1	918	1	0,829±0,026	100	4660
HMDH	HMG-CoA reductase	888	1	0,854±0,028	169	4668
HS90A	Heat shock protein HSP 90-alpha	732	1	0,849±0,027	84	4693
INHA	Enoyl-[acyl-carrier-protein] reductase	366	1	0,893±0,026	42	4694
KIF11	Kinesin-like protein 1	1056	1	0,878±0,025	115	4663
KITH	Thymidine kinase	234	1	0,893±0,027	56	4667
PA2GA	Phospholipase A2 group IIA	144	1	0,862±0,027	98	4661
PNPH	Purine nucleoside phosphorylase	289	1	0,879±0,029	102	4664
ROCK1	Rho-associated protein kinase 1	1354	1	0,943±0,020	99	4662
RXRA	Retinoid X receptor alpha	462	1	0,836±0,027	130	4719
SAHH	Adenosylhomocysteinase	432	1	0,828±0,026	62	4633
XIAP	Inhibitor of apoptosis protein 3	497	1	0,898±0,026	99	4669
ITAL	Leukocyte adhesion glycoprotein LFA-1α	1170	1	0,909±0,020	137	4639
PRGR	Progesterone receptor	933	1	0,868±0,025	292	4679
PYGM	Muscle glycogen phosphorylase	842	1	0,905±0,030	76	4666
ANDR	Androgen Receptor	920	0,9986	0,867±0,029	268	4641
PDE5A	Phosphodiesterase 5A	875	0,9956	0,913±0,026	397	4677
PYRD	Dihydroorotate dehydrogenase	395	0,9909	0,881±0,026	110	4674
MMP13	Matrix metalloproteinase 13	471	0,9901	0,897±0,027	571	4638
NOS1	Nitric-oxide synthase, brain	1434	0,9889	0,891±0,028	99	4692
CAH2	Carbonic anhydrase II	260	0,9863	0,892±0,029	491	4667
CASP3	Caspase-3	277	0,9795	0,913±0,030	198	4658
AKT2	Serine/threonine-protein kinase AKT2	481	0,9751	0,935±0,026	116	4673
FNTA	Protein farnesyltransferase type Iα subunit	379	0,9739	0,910±0,021	591	4663
AA2AR	Adenosine A2a receptor	412	0,9731	0,918±0,022	481	4719

Table 1. Model descriptors for 102 target proteins. The acronym, name, number of amino acids (AAs), PLA-Net model performance in average precision, average Rogot-Goldberg similarity between active ligands, number of active ligands and number of decoys are shown for each of the 102 target proteins.

Acronym	Target Protein	# of AAs	AP	Similarity train-test sets	Active ligands	Decoys
DRD3	Dopamine D3 receptor	400	0,9710	0,936±0,028	479	4657
DHI1	11-beta-hydroxysteroid dehydrogenase 1	292	0,9701	0,920±0,024	329	4663
BACE1	Beta-secretase 1	501	0,9603	0,900±0,028	282	4654
TRYB1	Tryptase beta-1	275	0,9597	0,933±0,028	147	4672
MK14	MAP kinase p38 alpha	360	0,9583	0,915±0,028	577	4647
CSF1R	Macrophage colony stimulating factor receptor	972	0,9550	0,912±0,031	165	4696
PARP1	Poly [ADP-ribose] polymerase-1	1014	0,9519	0,947±0,027	507	4670
NRAM	Neuraminidase	470	0,9514	0,816±0,026	97	4657
ADRB1	Beta-1 adrenergic receptor	477	0,9490	0,906±0,025	246	4699
PPARD	Peroxisome proliferator-activated receptor delta	441	0,9486	0,892±0,025	239	4660
HXK4	Hexokinase type IV	465	0,9477	0,920±0,025	91	4664
CDK2	Cyclin-dependent kinase 2	298	0,9396	0,927±0,024	473	4653
PGH2	Cyclooxygenase-2	604	0,9352	0,892±0,026	434	4700
ALDR	Aldose reductase	316	0,9271	0,892±0,024	158	4680
EGFR	Epidermal growth factor receptor erbB1	1210	0,9238	0,925±0,026	541	4668
ESR2	Estrogen receptor beta	530	0,9229	0,915±0,027	366	4689
PUR2	GAR transformylase	1010	0,9167	0,858±0,023	47	4705
SRC	Tyrosine-protein kinase SRC	536	0,9140	0,905±0,026	523	4619
PPARG	Peroxisome proliferator-activated receptor gamma	505	0,9136	0,895±0,025	483	4633
ESR1	Estrogen receptor alpha	595	0,9132	0,919±0,026	382	4671
VGFR2	Vascular endothelial growth factor receptor 2	1367	0,9114	0,924±0,024	408	4627
ACE	Angiotensin-converting enzyme	1306	0,9101	0,877±0,026	281	4577
WEE1	Serine/threonine-protein kinase WEE1	646	0,9049	0,925±0,022	101	4653
ACES	Acetylcholinesterase	614	0,8970	0,945±0,024	452	4665
HIVRT	Human immunodeficiency virus type 1 reverse transcriptase	259	0,8940	0,894±0,027	336	4675
PGH1	Cyclooxygenase-1	599	0,8912	0,887±0,026	194	4696
PTN1	Protein-tyrosine phosphatase 1B	435	0,8844	0,911±0,025	129	4672
HIVPR	Human immunodeficiency virus type 1 protease	100	0,8791	0,904±0,027	534	4661
PPARA	Peroxisome proliferator-activated receptor alpha	468	0,8771	0,893±0,023	372	4657
MCR	Mineralocorticoid receptor	984	0,8649	0,867±0,020	93	4645
LKHA4	Leukotriene A4 hydrolase	611	0,8608	0,963±0,028	170	4677

Table 1. Model descriptors for 102 target proteins. The acronym, name, number of amino acids (AAs), PLA-Net model performance in average precision, average Rogot-Goldberg similarity between active ligands, number of active ligands and number of decoys are shown for each of the 102 target proteins.

Acronym	Target Protein	# of AAs	AP	Similarity train-test sets	Active ligands	Decoys
THRB	Thrombin	622	0,8516	0,907±0,030	460	4659
ADRB2	Beta-2 adrenergic receptor	413	0,8490	0,906±0,028	230	4686
GRIA2	Glutamate receptor ionotropic, AMPA 2	883	0,8425	0,881±0,026	157	4658
TYSY	Thymidylate synthase	313	0,8319	0,864±0,029	108	4691
AKT1	Serine/threonine-protein kinase AKT	480	0,8271	0,936±0,023	292	4647
HDAC8	Histone deacetylase 8	377	0,8265	0,909±0,024	169	4627
MP2K1	Dual specificity mitogen-activated protein kinase kinase 1	393	0,8250	0,883±0,027	120	4678
CP3A4	Cytochrome P450 3A4	503	0,8110	0,910±0,025	169	4648
BRAF	Serine/threonine-protein kinase B-raf	766	0,7940	0,932±0,028	151	4675
HIVINT	Human immunodeficiency virus type 1 integrase	288	0,7852	0,882±0,028	99	4673
ABL1	Tyrosine-protein kinase ABL	1130	0,7850	0,920±0,022	181	4663
HDAC2	Histone deacetylase 2	488	0,7736	0,909±0,021	184	4660
FABP4	Fatty acid binding protein adipocyte	132	0,7615	0,888±0,023	46	4690
KIT	Stem cell growth factor receptor	976	0,7588	0,922±0,022	165	4633
MK10	c-Jun N-terminal kinase 3	464	0,7280	0,932±0,024	103	4668
RENI	Renin	406	0,7257	0,912±0,026	103	4658
KPCB	Protein kinase C beta	671	0,7183	0,935±0,024	134	4668
LCK	Tyrosine-protein kinase LCK	509	0,7100	0,920±0,015	419	4615
CP2C9	Cytochrome P450 2C9	490	0,7036	0,901±0,026	119	4671
FAK1	Focal adhesion kinase 1	1052	0,7022	0,917±0,024	99	4677
MET	Hepatocyte growth factor receptor	1390	0,6971	0,928±0,024	165	4686
AOFB	Monoamine oxidase B	520	0,6869	0,912±0,027	121	4699
FA10	Coagulation factor X	488	0,6838	0,908±0,021	536	4675
JAK2	Tyrosine-protein kinase JAK2	1132	0,6747	0,923±0,015	106	4650
DYR	Dihydrofolate reductase	187	0,6689	0,873±0,030	230	4656
PLK1	Serine/threonine-protein kinase PLK1	603	0,5963	0,890±0,025	106	4669
UROK	Urokinase-type plasminogen activator	431	0,5763	0,902±0,022	161	4671
MAPK2	MAP kinase-activated protein kinase 2	400	0,5737	0,949±0,029	100	4648
FGFR1	Fibroblast growth factor receptor 1	822	0,5550	0,909±0,029	138	4692
TGFR1	TGF-beta receptor type I	503	0,5427	0,948±0,025	132	4650
MK01	MAP kinase ERK2	360	0,5043	0,912±0,023	55	4693
TRY1	Trypsin I	247	0,5002	0,909±0,029	448	4616
THB	Thyroid hormone receptor beta-1	461	0,4177	0,868±0,027	102	4674
FA7	Coagulation factor VII	466	0,2720	0,895±0,017	113	4681
IGF1R	Insulin-like growth factor I receptor	1367	0,2331	0,930±0,019	147	4682

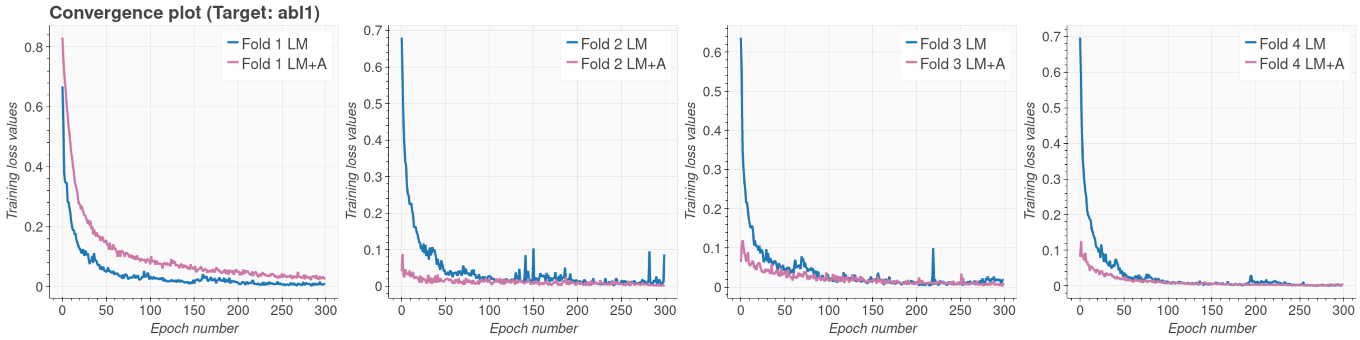
Convergence plot (Target: abl1)
Fold 1 LM
Fold 1 LM+A
Fold 2 LM
Fold 2 LM+A
Fold 3 LM
Fold 3 LM+A
Fold 4 LM
Fold 4 LM+A
Training loss values
Epoch number

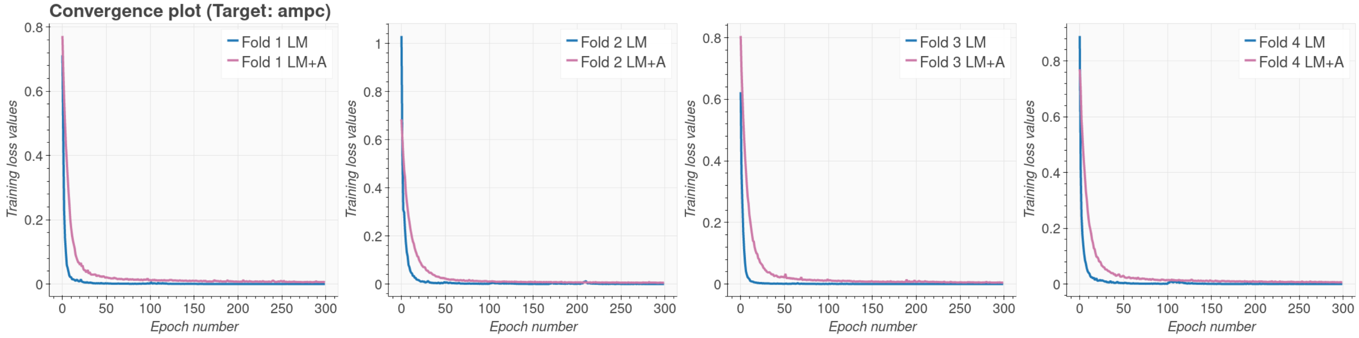
Convergence plot (Target: ampc)
Fold 1 LM
Fold 1 LM+A
Fold 2 LM
Fold 2 LM+A
Fold 3 LM
Fold 3 LM+A
Fold 4 LM
Fold 4 LM+A
Training loss values
Epoch number

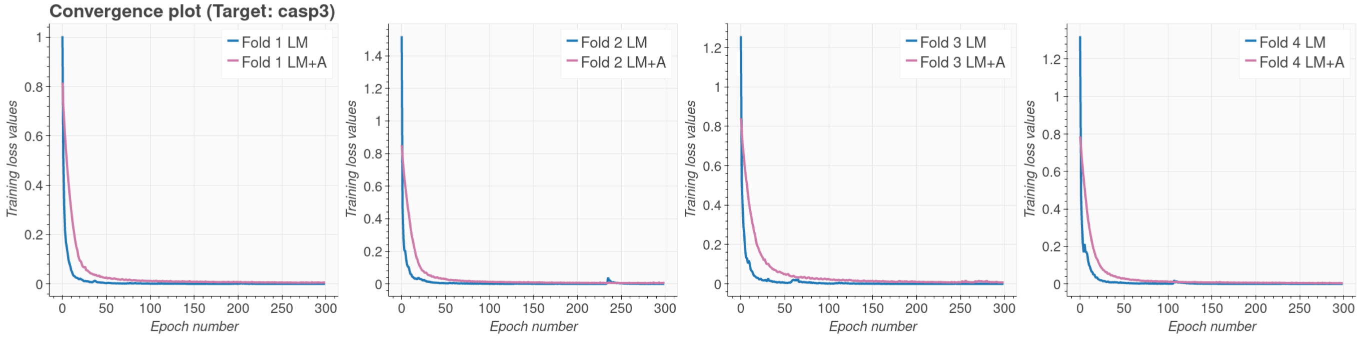
Convergence plot (Target: casp3)
Fold 1 LM
Fold 1 LM+A
Fold 2 LM
Fold 2 LM+A
Fold 3 LM
Fold 3 LM+A
Fold 4 LM
Fold 4 LM+A
Training loss values
Epoch number

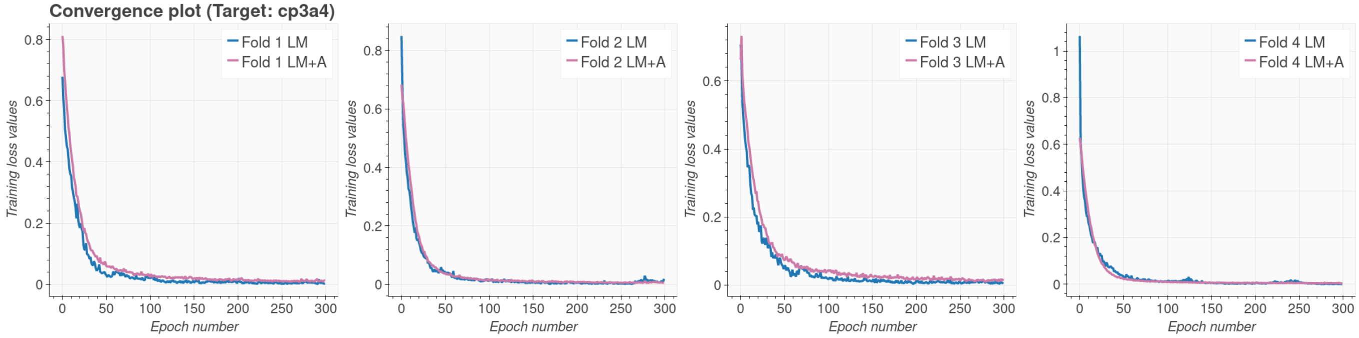
Convergence plot (Target: cp3a4)
Fold 1 LM
Fold 1 LM+A
Fold 2 LM
Fold 2 LM+A
Fold 3 LM
Fold 3 LM+A
Fold 4 LM
Fold 4 LM+A
Training loss values
Epoch number

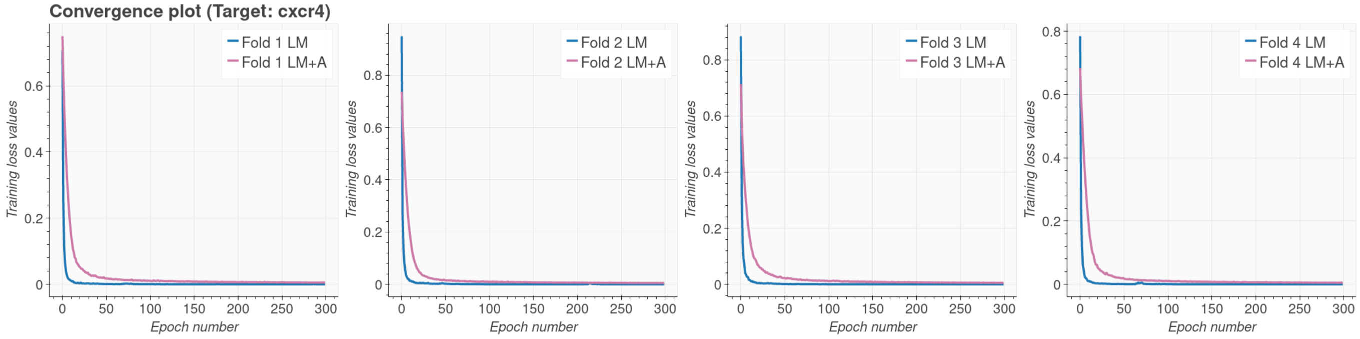
Convergence plot (Target: cxcr4)
Fold 1 LM
Fold 1 LM+A
Fold 2 LM
Fold 2 LM+A
Fold 3 LM
Fold 3 LM+A
Fold 4 LM
Fold 4 LM+A
Training loss values
Epoch number

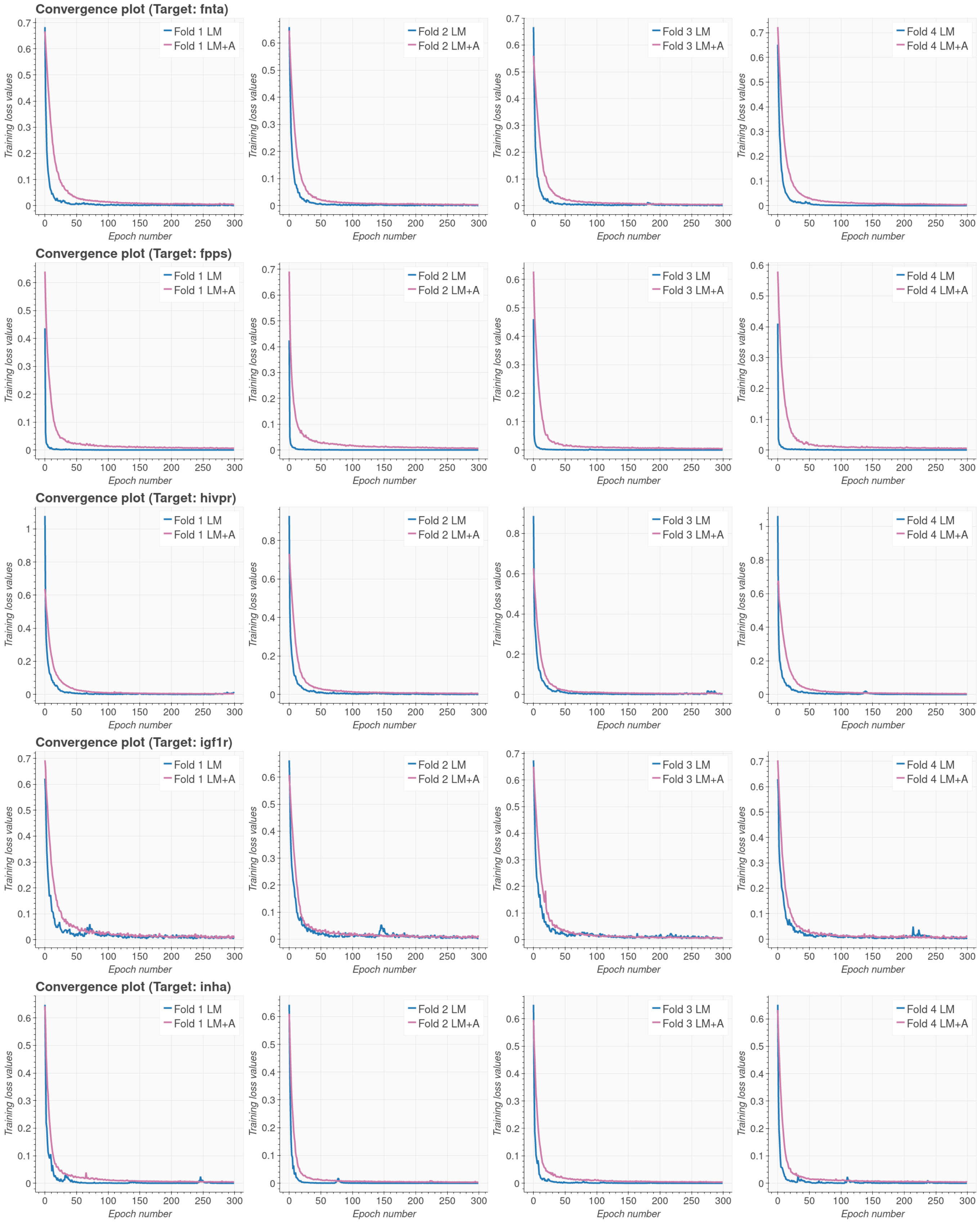
Convergence plot (Target: fnta)
Convergence plot (Target: fpps)
Convergence plot (Target: hivpr)
Convergence plot (Target: igf1r)
Convergence plot (Target: inha)
Fold 1 LM
Fold 1 LM+A
Fold 2 LM
Fold 2 LM+A
Fold 3 LM
Fold 3 LM+A
Fold 4 LM
Fold 4 LM+A
Training loss values
Epoch number

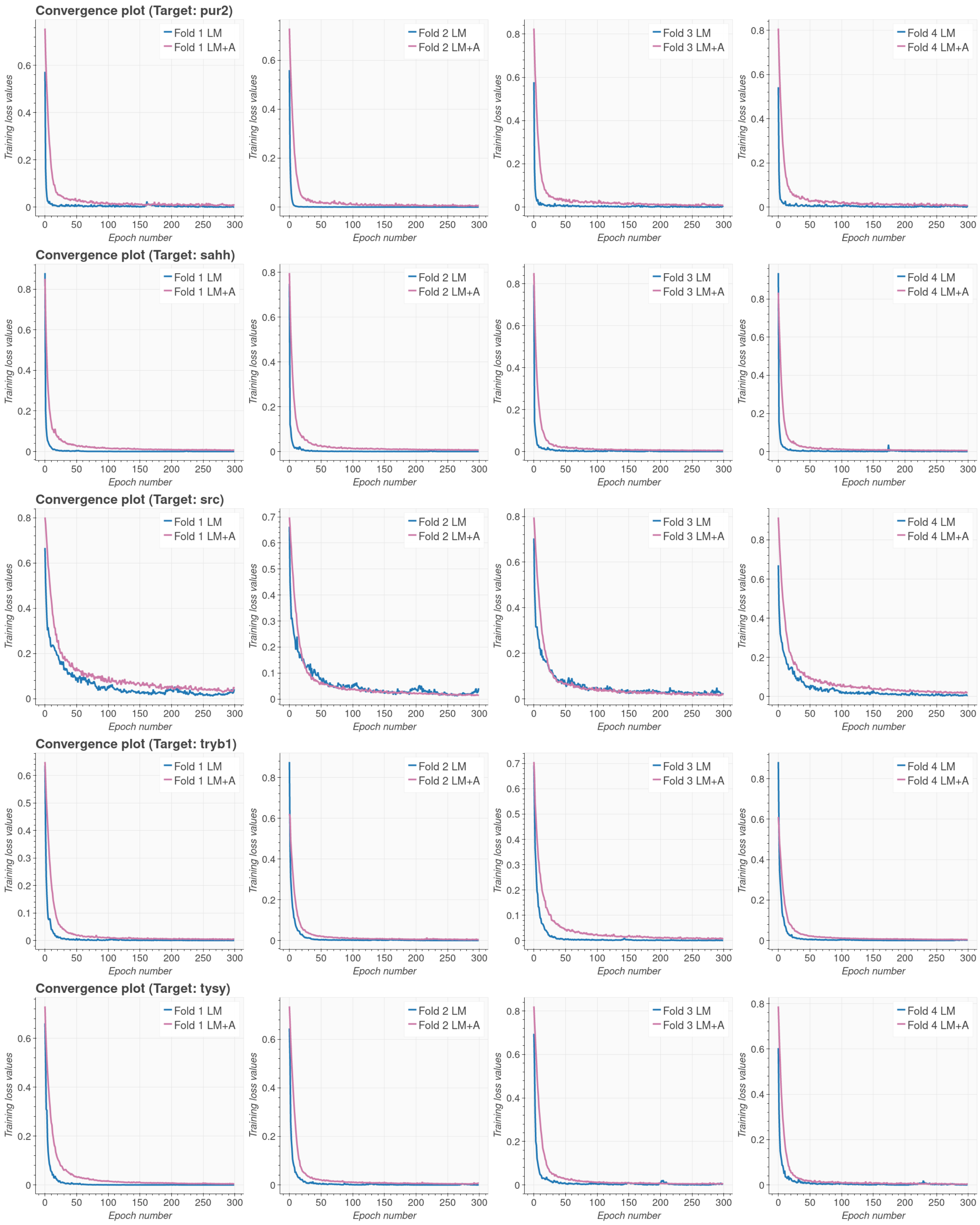

Figure 1. Convergence plots per fold of the LM and augmented LM of 15 representative targets. LM and augmented LM models usually converge during the first 100 epochs, with the LM converging slightly earlier in most cases.

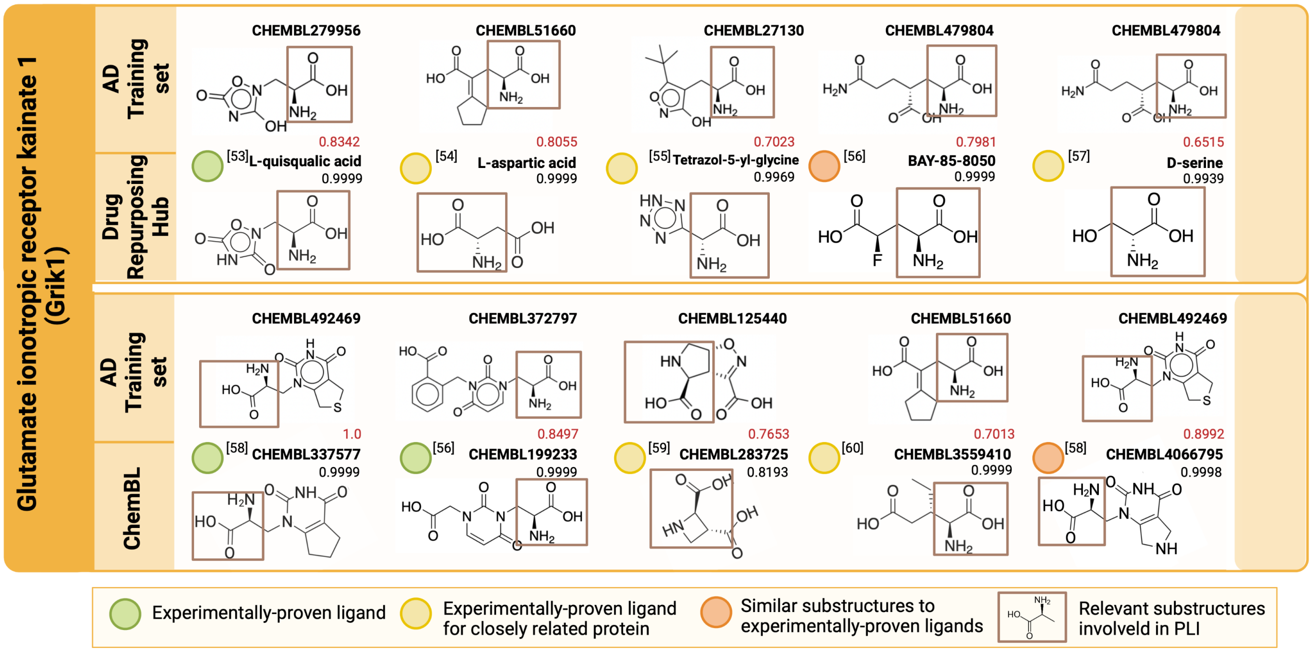

Figure 2. Comparison of predicted active molecules towards Grik1 from the Drug Repurposing Hub and CHEMBL datasets with the most similar active ligands in the AD training set. Fingerprint similarity between compared pairs is shown in red. Although some predicted ligands are closely respresented by active molecules in the AD training set (e.g., L-730 quiscalic acid, CHEMBL4066795), others that are not are still accurately identified by the model (e.g., CHEMBL199233, tetrazol-5-yl-glycine, CHEMBL283725).
